# Targeting 160 Candidate Genes for Blood Pressure Regulation with a Genome-Wide Genotyping Array

**DOI:** 10.1371/journal.pone.0006034

**Published:** 2009-06-29

**Authors:** Siim Sõber, Elin Org, Katrin Kepp, Peeter Juhanson, Susana Eyheramendy, Christian Gieger, Peter Lichtner, Norman Klopp, Gudrun Veldre, Margus Viigimaa, Angela Döring, for the Kooperative Gesundheitsforschung in der Region Augsburg study, Margus Putku, Piret Kelgo, for the HYPertension in ESTonia study, Sue Shaw-Hawkins, Philip Howard, Abiodun Onipinla, Richard J. Dobson, Stephen J. Newhouse, Morris Brown, Anna Dominiczak, John Connell, Nilesh Samani, Martin Farrall, for the MRC British Genetics of Hypertension study, Mark J. Caulfield, Patricia B. Munroe, Thomas Illig, H.-Erich Wichmann, Thomas Meitinger, Maris Laan

**Affiliations:** 1 Institute of Molecular and Cell Biology, University of Tartu, Tartu, Estonia; 2 Institute of Epidemiology, Helmholtz Zentrum München, German Research Centre for Environmental Health, Neuherberg, Germany; 3 Department of Statistics, Pontificia Universidad Catolica de Chile, Santiago, Chile; 4 Institute of Human Genetics, Helmholtz Zentrum München, German Research Centre for Environmental Health, Neuherberg, Germany; 5 Department of Cardiology, University of Tartu, Tartu, Estonia; 6 Centre of Cardiology, North Estonia Medical Centre, Tallinn, Estonia; 7 Clinical Pharmacology and The Genome Centre, William Harvey Research Institute, Barts and The London School of Medicine and Dentistry, London, United Kingdom; 8 Clinical Pharmacology Unit, University of Cambridge, Addenbrookes Hospital, Cambridge, United Kingdom; 9 Glasgow Cardiovascular Research Centre, University of Glasgow, Glasgow, United Kingdom; 10 Cardiovascular Sciences, University of Leicester, Glenfield Hospital, Leicester, United Kingdom; 11 Cardiovascular Medicine, University of Oxford, Wellcome Trust Centre for Human Genetics, Oxford, United Kingdom; 12 Institute of Medical Informatics, Biometry and Epidemiology, Ludwig-Maximilians-Universität, Munich, Germany; 13 Institute of Human Genetics, Klinikum rechts der Isar, Technical University of Munich, Munich, Germany; University of Wuerzburg, Germany

## Abstract

The outcome of Genome-Wide Association Studies (GWAS) has challenged the field of blood pressure (BP) genetics as previous candidate genes have not been among the top loci in these scans. We used Affymetrix 500K genotyping data of KORA S3 cohort (n = 1,644; Southern-Germany) to address (i) SNP coverage in 160 BP candidate genes; (ii) the evidence for associations with BP traits in genome-wide and replication data, and haplotype analysis. In total, 160 gene regions (genic region±10 kb) covered 2,411 SNPs across 11.4 Mb. Marker densities in genes varied from 0 (n = 11) to 0.6 SNPs/kb. On average 52.5% of the HAPMAP SNPs per gene were captured. No evidence for association with BP was obtained for 1,449 tested SNPs. Considerable associations (*P*<10^−3^) were detected for the genes, where >50% of HAPMAP SNPs were tagged. In general, genes with higher marker density (>0.2 SNPs/kb) revealed a better chance to reach close to significance associations. Although, none of the detected *P*-values remained significant after Bonferroni correction (*P*<0.05/2319, *P*<2.15×10^−5^), the strength of some detected associations was close to this level: rs10889553 (*LEPR*) and systolic BP (SBP) (*P* = 4.5×10^−5^) as well as rs10954174 (*LEP*) and diastolic BP (DBP) (*P* = 5.20×10^−5^). In total, 12 markers in 7 genes (*ADRA2A, LEP, LEPR, PTGER3, SLC2A1, SLC4A2, SLC8A1*) revealed considerable association (*P*<10^−3^) either with SBP, DBP, and/or hypertension (HYP). None of these were confirmed in replication samples (KORA S4, HYPEST, BRIGHT). However, supportive evidence for the association of rs10889553 (*LEPR*) and rs11195419 (*ADRA2A)* with BP was obtained in meta-analysis across samples stratified either by body mass index, smoking or alcohol consumption. Haplotype analysis highlighted *LEPR* and *PTGER3*. In conclusion, the lack of associations in BP candidate genes may be attributed to inadequate marker coverage on the genome-wide arrays, small phenotypic effects of the loci and/or complex interaction with life-style and metabolic parameters.

## Introduction

Hypertension is considered a multifactorial disease – it is influenced by multiple genetic loci as well as environmental and lifestyle factors including diet, smoking habits and physical exercise. Despite the relatively high heritability (*h^2^* 0.3–0.6) of blood pressure [1], the identification of genetic factors contributing to the variation in blood pressure levels and susceptibility to hypertension in general population has remained challenging. Majority of the currently available knowledge has been gained by mapping the genes responsible for Mendelian forms of hyper- and hypotension [2] and studying rodent models with various blood pressure affecting phenotypes (e.g. Spontaneously hypertensive rats) [3], [4]. These studies have successfully pinpointed multiple interacting molecular pathways that are involved in the determination of a subject's blood pressure. Consequently, the genes coding for the components of these molecular pathways have been targets for the identification of common and rare genetic variation affecting inter-individual differences in blood pressure levels [2], [5], [6]. However, the results of a large number of association studies conducted with blood pressure traits have been inconsistent, suggesting a high locus and inter-population heterogeneity and trait complexity. Also, differences in recruitment strategies between the original and replication studies may affect the results [7].

Genome wide association (GWA) based studies have emerged as a novel alternative to explore simultaneously a large number of genomic loci for associations with a phenotypic trait. This method has shown great promise in ascertaining common polymorphisms responsible for several complex phenotypes like diabetes, stroke and coronary artery disease [8]. However, GWA studies with blood pressure have not produced unequivocal results [7], [9], [10]. Disappointingly, the previously studied candidate genes have not been among the top loci in these association scans. This outcome of GWA studies has challenged several decades of research in the field of blood pressure physiology and genetics that has produced a large quantity of data implicating many genes in various aspects of blood pressure regulation. One explanation may be that blood pressure is determined by the combination of a large number of gene variants, each contributing with a small effect, and thus the conducted studies have been underpowered. Further possibility is that blood pressure candidate genes have been insufficiently tagged on the genotyping arrays [5], [11], [12].

We used Affymetrix 500K as model for a genome-wide genotyping array and analyzed the genotyping data of 1,644 individuals from the Kooperative Gesundheitsforschung in der Region Augsburg (KORA) S3 cohort originating from Southern-Germany to study the following questions: (i) How are 160 genes with prior evidence for involvement in blood pressure regulation covered on the Affymetrix 500K array? (ii) Which candidate genes show the strongest signal for association in an evidence-based association study addressed by a genome-wide dataset? Are the detected associations replicable in other study samples and meta-analysis across samples? (iii) Does haplotype analysis improve the results obtained for single markers?

## Materials and Methods

### Selection of candidate genes

The analysis focused on 160 candidate genes with prior evidence of being involved in blood pressure regulation (**Table S1** **,** **Table S2**). Majority of the genes were selected from published literature reports on biology and genetics of blood pressure. Selection criteria included genes responsible for the Mendelian forms of hypertension or hypotension, location near linkage peaks or quantitative trait loci (QTLs), reports on animal models and human association studies etc. In addition to literature, additional information was obtained from various genome resources (OMIM, http://www.ncbi.nlm.nih.gov/sites/entrez?db=omim; NCBI GeneBank and NCBI Locuslink http://www.ncbi.nlm.nih.gov/; Ensembl http://www.ensembl.org/index.html). Candidate gene list was also supplemented with loci involved in other cardiovascular diseases related to hypertension (myocardial infarction, coronary artery disease and stroke).

Studied genomic regions were defined to include the candidate genes ±10 kb of flanking sequence around each gene (defined according to NCBI Build 35).

### Ethics Statement

A detailed description of the recruitment and the applied standardized examinations of the discovery sample KORA S3 (Kooperative Gesundheitsforschung in der Region Augsburg) and replication samples KORA S4, HYPEST (HYPertension in ESTonia) and BRIGHT (The MRC British Genetics of Hypertension) has been described elsewhere [13], [14], [15]. All studies have been approved by the local ethics committees and all participants have given written informed consent.

### Study subjects

All participants from KORA, HYPEST and BRIGHT studies are of white European ancestry. The demographics of each study sample is given in **Table S3**.

Briefly, KORA S3 and KORA S4 epidemiological cohorts represent independent samples of unrelated subjects from the general population from the Augsburg Area (Southern Germany) recruited in 1994–1995 (S3) and 1999–2001 (S4) [14]. KORA individuals (n = 1,644) with data available for S3 and F3 (the follow-up recruitment of S3 participants 10 years later, in 2003–2004) surveys were selected for genome-wide genotyping. HYPEST study sample consists of unrelated subjects (n = 1,823) recruited between 2004 and 2007 across Estonia with the aim to identify hypertension risk factors in the Estonian population [15]. The recruited individuals have detailed epidemiological data and a documented history of multiple systolic (SBP) and diastolic (DBP) blood pressure measurements (on average 4.31 readings per individual) during mean 3.17 years. The BRIGHT case-control samples have been recruited across United Kingdom (UK) (http://www.brightstudy.ac.uk)[13]. Cases originated from severely hypertensive families (1700 sib-pairs and 800 nuclear families) and were defined as patients under antihypertensive treatment and with BP readings ≥150/100 mmHg based on one reading or ≥145/95 mmHg based on the mean of three readings. Healthy normotensive controls (n = 2000; blood pressure<140/90 mmHg, no antihypertensive medication and no diagnosed diseases) were recruited by matching age, sex and geographical distribution across UK.

### Genome-wide single-nucleotide polymorphism (SNP) data and genotyping in replication experiments

Genome-wide SNP genotyping of 1,644 KORA S3 samples was carried out in the framework of the KORA 500K Consortium (http://epi.helmholtz-muenchen.de/kora-gen/seiten/kora500k_e.php). Genotyping was performed using the Affymetrix Gene Chip Human Mapping 500K Array Set consisting of two chips (Sty I and Nsp I) according to the manufacturer's instructions. The genotypes were determined using BRLMM clustering algorithm. Genotyping laboratory experiments, call of genotypes and genotyping quality control is described in detail elsewhere (http://epi.gsf.de/kora-gen/seiten/kora500k_e.php). SNPs with signals of unreliable genotyping quality (call rate<93%) and deviation from Hardy-Weinberg Equilibrium (HWE; p<0.001) were excluded from statistical analysis.

Replication genotyping of seven SNPs in KORA S4 samples (n = 1,830) was performed using the iPLEX assay (Sequenom). After applying a call rate cutoff (93%) n = 1765 individuals entered the statistical analysis. Genotyping of two SNPs (rs10889553, rs11195419) in the HYPEST and the BRIGHT samples was performed using the KASPar chemistry, a competitive allele specific PCR SNP genotyping system using FRET quencher cassette oligos (Genotyping Unit of the William Harvey Research Institute). Call rate was >97% for the HYPEST and >95% for the BRIGHT samples. Both SNPs genotyped in replication stage were in HWE.

### Phenotypes in association analysis of Affymetrix 500K SNPs in hypertension candidate genes and in replication steps

Association analysis of Affymetrix 500K SNPs in hypertension candidate genes was carried out using the KORA S3 500K Consortium dataset (genotyped subjects: n = 1,644; body mass index, BMI = 27.7±4.2 kg/m^2^; age = 55.6±7.0 years). Association with systolic (SBP) and diastolic (DBP) blood pressure was tested using only individuals, who were not receiving blood pressure lowering medication (n = 1,017). For case-control analysis the groups of hypertensives (n = 364) was defined as: (i) individuals under antihypertensive medication; (ii) untreated subjects with SBP≥160 mmHg and/or DBP≥100 (Grade 2 hypertension) mmHg or (iii) untreated individuals with SBP≥140 mmHg (Grade 1 hypertension) in S3 that developed 10 years later to Grade 2 or severe hypertension (F3 survey). Control subjects (n = 596) were selected to have optimal (<120/80 mmHg) or normal (<130/85 mmHg) blood pressure measured during both S3 (original) and F3 (ten years later) surveys, and had never been prescribed antihypertensive medication. For replication we selected the individuals from KORA S4 survey (n = 1,830; BMI = 25.6±2.7 kg/m^2^; age = 52.7±9.0 years) that satisfied the same phenotype criteria as in GWAS with KORA S3. A total of 1,551 subjects entered the association analysis with SBP/DBP. Case-control association analysis (HYP) was performed with 447 hypertensives/1,119 normotensives.

Further replication was performed with subjects from the HYPEST (total n = 1,823; BMI = 26.2±3.9 kg/m^2^; age = 45.4±13.7 years) and BRIGHT (total n = 4,370; BMI = 26.6±3.8 kg/m^2^; age = 58.3±10.1 years) studies. Association analysis with SBP and DBP was performed using 1,097 untreated HYPEST individuals. In HYPEST, the association analysis with hypertension (HYP) was performed with cases (n = 596) defined as subjects with either BP readings≥160/100 mm Hg based on the median of several measurements or receiving anti-hypertensive therapy. Controls (n = 650; blood pressure<130/85 mm Hg based on mean of two independent readings across mean 3.17 years or<140/90 mm Hg based on mean of ≥3 readings) were selected among the subjects that have never been prescribed antihypertensive treatment. Case-control association analysis (HYP) in the BRIGHT study was performed with 2,401 hypertensives/1,969 normotensives. Since BRIGHT cases (severe hypertension; all subjects treated with anti-hypertensive medication) were collected as extremes, we were unable to test associations with SBP and DBP.

### Statistical methods

Testing for single marker associations was performed using PLINK software (http://pngu.mgh.harvard.edu/~purcell/plink/) [16] version 0.99q. Binary phenotype data was analysed using Cochran-Armitage trend test (1 degree of freedom) and multivariate analysis was performed using logistic regression with additive disease model (age and sex of individuals were included as covariates). SBP and DBP were tested using linear regression with and without covariates (age, sex). No minor allele frequency limit was set for the used markers and all markers that passed quality control were tested for association.

The contribution of life-style factors to the association of genetic markers with blood pressure was assessed by reanalyzing the study samples stratified by: (i) body mass index, BMI [normal weight, BMI<25 kg/m^2^ (meta-analysis for SBP&DBP n = 1,420, for HYP n = 2,532); overweight, BMI≥25 kg/m^2^ (meta-analysis for SBP&DBP n = 2,423, for HYP n = 4,859)]; (ii) smoking habits [non-smokers (meta-analysis for SBP&DBP n = 1,558, for HYP n = 1,724); smokers, including smokers at the recruitment and ex-smokers (meta-analysis for SBP&DBP n = 1,708, for HYP n = 1,723)] and (iii) alcohol consumption [low consumers, <20 g/day (meta-analysis for SBP&DBP n = 2,054, for HYP n = 2,222); high consumers, ≥20 g/day (meta-analysis for SBP&DBP n = 1,205, for HYP n = 1,218)]. For the BRIGHT study participants the data for smoking and alcohol consumption was not available.

For analysing haplotype effects we used the WHAP version 2.09 software (http://pngu.mgh.harvard.edu/~purcell/whap/) [17], which implicitly performs phasing and accounts for ambiguity in haplotype inference. Regression based omnibus tests were performed for all haplotypes with >1% frequencies at a given locus. We used sliding window testing with corrections for multiple testing within each studied region. Sliding window sizes of 1, 3, 5, and 8 SNPs with a step size of 1 marker were used in our study. 600 permutations of data were performed for each window to generate empirical P values. For detailed analysis and visualization of results we used PHASE 2.1 software with default parameters to generate estimated haplotypes for each individual in our sample [18], [19]. Haplotype effects to SBP were tested using two tailed Mann-Whitney U test in R statistical package (http://www.r-project.org/) [20] and haplotype effects to hypertension phenotype were tested with logistic regression in PLINK. Haploview 4.0 [21] was used to generate LD plots (http://www.broad.mit.edu/mpg/haploview/index.php). Coverage was measured as the percentage of SNPs (minor allele frequency, MAF≥0.05) in Hapmap phase II (release 23; CEU population) (http://www.hapmap.org/) [22] localized in genic regions that were tagged on the Affymetrix 500 k chip with r^2^≥0.8. The analysis included SNPs on the Affymetrix array within ±200 kb from a gene and passing quality control criteria in KORA 500K genotyping. Meta analysis of studied populations was performed in R (meta package) using inverse variance method with a fixed effect model. Power calculations for linear regressions to SBP were performed in R (pwr package). SBP distribution data from KORA S3 was used to estimate f^2^ values assuming additive genotype effects.

## Results

### Blood pressure candidate genes are unevenly covered with SNPs on Affymetrix 500K array

**Figure 1:**
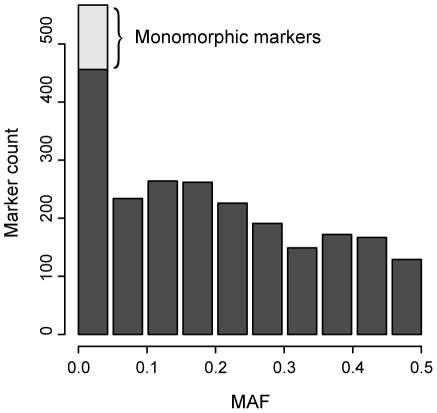
Histogram of minor allele frequencies of Affymetrix 500K markers (n = 2411) located in blood pressure candidate gene regions. Monomorphic markers (n = 111) are indicated in light gray colour.

The study was focused on Affymetrix 500K array SNPs tagging genomic regions of 160 genes with prior evidence for the involvement in blood pressure regulation (**Table S1** **,** **Table S2**). In total, the genomic regions entering the analysis (genic region ±10 kb) covered 11.4 Mb and contained 2411 genotyped markers (**Table 1**). SNPs were distributed across intronic (n = 1555; 64%), exonic (n = 387; 16%) and flanking intergenic regions (n = 488; 20%). The analysis included also 21 non-synonymous changes. In genotyped KORA S3 population, a notable fraction of SNPs were either monomorphic (n = 107; 4.4%) or rare (minor allele frequency, 0%<MAF<5%) variants (n = 464, 19.2%; **Figure 1**).

**Table 1:** Affymetrix 500K SNPs (n = 2,411) in blood pressure candidate genes by location and functional category.

Marker category	No of markers
intronic	1555 (65%)
exonic	387 (16%)
untranslated regions	329 (85%)
coding regions	58 (15%)
non-synonymous	21 (36%)
synonymous	37 (64%)
intergenic	488 (19%)

**Figure 2:**
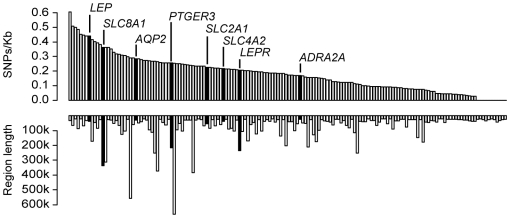
Coverage of blood pressure candidate genes (genic region ±10 kb) with Affymetrix 500K chip SNP markers. Upper and lower parts of the graph depict SNP density/kb and length of each analyzed gene region, respectively. Genes harboring the SNPs, which resulted in strongest associations (*P*<10^−3^; detailed in Table 2) with hypertension (HYP), systolic (SBP) or diastolic (DBP) blood pressure in KORA S3 cohort are highlighted in black.

The length of the studied gene regions in Affymetrix 500K array ranged from 0.8 kb (*NPPC*) to 645 kb (*CACNA1C*) (**Table S1** **,** **Table S2**). The mean number of genotyped SNP-s/gene was 15.2 (range: 0–168) with the average intermarker distance 6.5 kb. Marker densities varied from 0 to 0.6 markers/kb. A total of 11 (7%) short genes (≤23 kb) did not contain any Affymetrix 500K markers and had to be excluded from the analysis (*ADRB3, AVP, AVPR1B, GIPR, HP, INS, SLC5A2, SLC9A5, TBXA2R, TH* and *VEGFB*; **Figure 2**). Genes with higher marker density (>0.2 SNPs/kb) had a better chance to exhibit putative associations (*P*<10^−3^) with tested blood pressure traits (**Figure 2**). The strongest associations in genes with low marker density (n = 74; 46% of genes; 0–0.16 SNPs/kb) did not reach *P*<10^−3^.

We further explored the representation of the HAPMAP SNPs within blood pressure candidate genes on the Affymetrix 500K array. On average 52.5% of the HAPMAP SNPs per gene were captured (median coverage 59.5%) (**Table S1** **,** **Table S2**). Putative associations with blood pressure traits (*P*<10^−3^) were detected for the genes, where >50% of HAPMAP SNPs were tagged. The one exception was *ADRA2A* (coverage 33.3%). For 71 genes (44.4%) Affymetrix 500K array captured only up to 50% of the HAPMAP variants including 31 genes (19.4%) not tagged at all. Overall we found that marker densities and coverage on the Affymetrix 500K SNP-array are sufficient to test associations with only half of the selected genes.

### Single marker association analysis of GWA data

Affymetrix 500K genotype data for 2,304 polymorphic SNPs located in 149 blood pressure candidate genes was derived from the KORA S3 500K Consortium dataset (n = 1,644).

**Figure 3:**
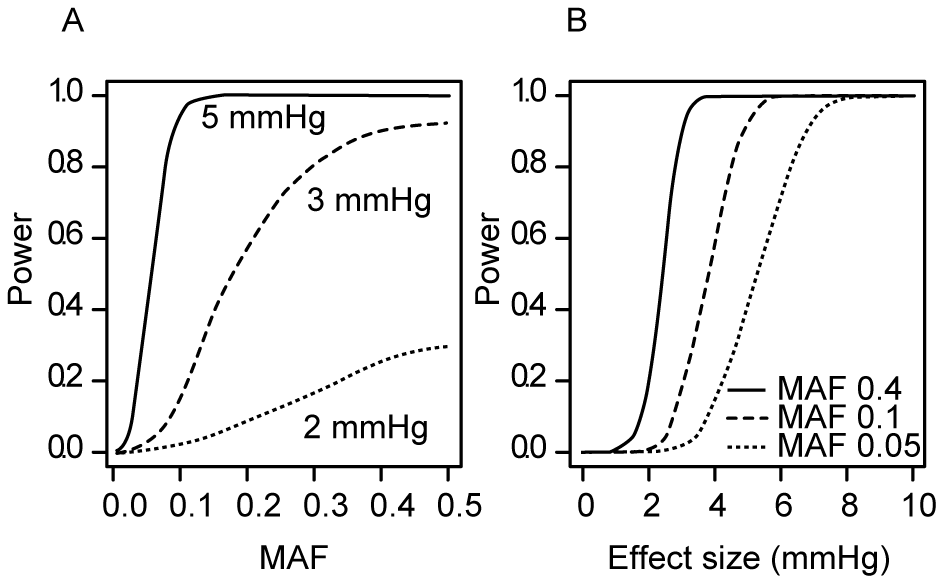
Power analysis. Calculated statistical power at the Bonferroni significance level (α = 2.15×10^−5^) for the detection of SNP effects on SBP in the discovery sample KORA S3 (untreated subjects, n = 1,017). Power according to (A) alternative minor allele frequencies (MAF), shown for different effect sizes (2, 3, 5 mmHg) and (B) effect sizes, shown for different MAFs (5%, 10%, 40%).

Associations with hypertension (HYP) were tested by trend test and logistic regression under additive genetic model adjusted by age and sex as covariates. Associations with systolic (SBP) and diastolic (DBP) blood pressure were assessed by linear regression. More than half of the tested Affymetrix 500K markers (n = 1449 markers; 63%) covering the majority of the candidate gene regions (n = 130; 81%) showed no evidence for association with blood pressure (BP) in KORA S3 study sample (**Table S2**). Only 12 (0.50% of total tested) markers in 7 genes (*ADRA2A, LEP, LEPR, PTGER3, SLC2A1, SLC4A2, SLC8A1*) revealed close to significance associations (*P*<10^−3^) with at least one blood pressure trait (**Table 2**). Additional 94 (4.1%) markers in 26 genes exhibited suggestive associations with 0.01<*P*<0.001 (**Table S1**). The strongest associations were detected between rs10954174 in *Leptin* (*LEP*) gene and DBP (*P* = 5.2×10^−5^, linear regression); between rs10889553 in *Leptin receptor gene* (*LEPR*) and SBP (*P* = 4.5×10^−5^, linear regression) as well as hypertension (*P = *4.14×10^−4^, logistic regression). Although, none of the detected *P* values remained significant after Bonferroni correction (*P*<0.05/2319, *P*<2.15×10^−5^), the strength of some of the detected associations was close to this level. Power analysis revealed that the discovery cohort KORA S3 was underpowered for the detection of associations with markers with low minor allele frequency (MAF<0.1) (**Figure 3A**) and/or small effect size (<3 mmHg for SBP) (**Figure 3B**).

**Table 2:** The strongest associations (*P*<10^−3^) between blood pressure traits and Affymetrix 500K chip SNPs located in hypertension candidate genes.

Gene		Length	SNPs Affy on	Average SNP				SBP	DBP	Hypertension	
Symbol	Chr	(bp)*	500K	distance	Top SNPs			Linear regression	Linear regression	Trend test	Logistic Regression
					rs number	MAF	Location	*P-*value (beta)	*P-*value (beta)	*P-*value	*P-*value (OR)
ADRA2A	10	23649	4	5.9 kb	rs11195419	10.6%	3′ UTR	3.98×10^−2^ (2.57)	**6.56×10^−4^ (2.56)**	4.80×10^−2^	1.52×10^−2^ (1.50)
					rs11195417	9.4%	upstream	2.58×10^−2^ (3.00)	**8.31×10^−4^ (2.71)**	2.48×10^−2^	7.61×10^−3^ (2.68)
*LEP*	7	36350	16	2.3 kb	rs10954174	0.1%	3′ UTR	4.48×10^−2^ (23.6)	**5.20×10^−5^ (28.6)**	7.42×10^−2^	1.00 (0.001)
*LEPR*	1	236502	49	4.8 kb	rs10889553	5.4%	intron	**4.50×10^−5^ (6.61)**	3.16×10^−3^ (2.90)	**1.61×10^−4^**	**4.14×10^−4^ (2.16)**
					rs17097182	5.2%	intron	**6.68×10^−4^ (5.62)**	1.98×10^−2^ (2.33)	**7.13×10^−4^**	4.14×10^−3^ (1.88)
*PTGER3*	1	215455	55	3.9 kb	rs2268062	38.1%	intron	4.27×10^−2^ (−1.51)	0.15 (−0.65)	**8.10×10^−4^**	7.21×10^−3^ (−2.69)
*SLC2A1*	1	52982	12	4.4 kb	rs1105297	31.7%	intron	0.58 (−0.49)	**8.27×10^−4^ (−1.67)**	0.39	0.23 (−1.20)
*SLC4A2*	7	36870	8	4.6 kb	rs2303934	3.2%	intron	0.24 (2.82)	0.36 (1.32)	**5.66×10^−4^**	1.38×10^−3^ (3.21)
*SLC8A1*	2	338158	123	2.7 kb	rs405884	9.8%	intron	**2.27×10^−4^ (−4.82)**	2.49×10^−2^ (−1.75)	**3.90×10^−4^**	**3.13×10^−4^ (−3.62)**
					rs406222	8.2%	intron	1.48×10^−3^ (−4.15)	2.06×10^−3^ (−2.41)	**6.62×10^−4^**	1.22×10^−3^ (−3.24)
					rs394112	8.4%	intron	1.48×10^−3^ (−4.18)	4.01×10^−3^ (−2.29)	**8.43×10^−4^**	**9.00×10^−4^ (−3.33)**
					rs415695	9.5%	intron	1.86×10^−3^ (−3.86)	1.11×10^−2^ (−1.9)	1.66×10^−3^	**9.60×10^−4^ (−3.31)**

* Length of the analyzed region includes genic region ±10 kb. *P*-values<10^−3^ are indicated in bold. Hypertension – hypertensives/normotensives as cases and controls; Chr – chromosome; MAF – minor allele frequency; OR – Odds Ratio.

In three genes evidence for association with BP (*P*<10^−3^) was detected with multiple markers: intronic SNPs in *LEPR* and *Sodium-Calcium Exchanger 1 (SLC8A1)* were associated with SBP and HYP; and markers localized in *Alpha-2A-Adrenergic Receptor* (*ADRA2A*) intronless gene region were associated with DBP (**Table 2**).

### Replication of the initial associations and combined analysis of the data

One SNP from each of the seven loci (*ADRA2A, LEP, LEPR, PTGER3, SLC2A1, SLC4A2, SLC8A1*) revealing an association with blood pressure traits in the KORA S3 discovery sample was selected for replication. First replication was attempted using KORA S4 sample (n = 1,830), an independently recruited cohort from the same population (Southern-Germany). None of the SNPs entering replication retained their initial significance level of *P*<10^−3^. Four SNPs (rs10889553, rs2303934, rs10954174, rs11195419) were found to be marginally associated with BP traits with *P* values<0.1 (**Table S4**). Two of these markers (rs10889553 and rs11195419) with consistent effects in KORA S3 and KORA S4 were selected for further replication in HYPEST (Estonian) cohort and in BRIGHT (UK) hypertensives-normotensives case-control sample set. As in KORA S4, no statistically significant association was detected between the studied SNPs in replication samples HYPEST and BRIGHT (**Table S4**), as well as in the meta-analysis combining the results of all replication resources (n = 3,638 for SBP and DBP, n = 7,553 for HYP, **Table 3**). However, in the joint meta-analysis of the KORA S3 data and the replication samples the strength of the association between rs10889553 (*LEPR*) and DBP was enhanced compared to the original signal (discovery sample KORA S3, *P* = 3.16×10^−3^, beta = 2.79; Meta-analysis *P* = 2.4×10^−3^, beta = 1.51).

**Table 3:** The associations of rs10889553 and rs11195419 with blood pressure in the discovery sample KORA S3, replication dataset and meta-analysis: results for the full study sample and sub-samples stratified based on lifestyle factors.

Analysis	cohort		rs10889553	(*LEPR*)		rs11195419	(*ADRA2A*)	
group			SBP	DBP	HYP	SBP	DBP	HYP
All subjects	KORA S3	P value	4.50×10^−5^	3.16×10^−3^	4.14×10^−4^	3.98×10^−2^	6.56×10^−4^	9.32×10^−3^
		beta	6.43	2.79	0.75	2.83	2.62	0.41
		95% CI	3.29; 9.57	0.89; 4.69	0.32; 1.17	0.47; 5.19	1.20; 4.04	0.10; 0.73
	Replication^a^	P value	0.37	6.66×10^−2^	0.43	0.53	0.59	0.12
		beta	0.84	1.06	0.07	0.49	0.26	0.10
		95% CI	−1.00; 2.69	−0.07; 2.20	−0.11; 0.24	−1.06; 2.04	−0.69; 1.22	−0.02; 0.22
	KORA S3 and	P value	5.60×10^−3^	2.4×10^−3^	4.52×10^−2^	8.89×10^−2^	1.39×10^−2^	1.74×10^−2^
	replication^b^	beta	2.25	1.51	0.16	1.12	1.03	0.14
		95% CI	0.66; 3.85	0.53; 2.49	0.00; 0.32	−0.17; 2.42	0.20; 1.78	0.02; 0.25
*Stratified by BMI:*								
BMI≥25 kg/m^2^	KORA S3	P value	6.41×10^−3^	1.70×10^−2^	1.89×10^−2^	7.97×10^−3^	1.57×10^−4^	5.21×10^−2^
		beta	4.84	2.51	0.49	3.63	3.06	0.31
		95% CI	1.37; 8.31	0.45; 4.57	0.08; 0.90	0.96; 6.31	1.48; 4.64	0.00; 0.63
	Replication^a^	P value	0.41	0.96	0.48	0.13	0.34	4.48×10^−2^
		beta	−1.04	0.04	0.07	1.59	0.60	0.16
		95% CI	−3.50; 1.42	−1.42; 1.50	−0.13; 0.28	−0.46; 3.65	−0.62; 1.81	0; 0.31
	KORA S3 and	P value	0.37	0.15	9.23×10^−2^	6.90×10^−3^	2.10×10^−3^	8.00×10^−3^
	replication^b^	beta	0.92	0.87	0.16	2.44	1.51	0.18
		95% CI	−1.08; 2.93	−0.32; 2.06	−0.03; 0.34	0.67; 4.20	0.55; 2.47	0.05; 0.32
BMI<25 kg/m^2^	KORA S3	P value	5.16×10^−2^	0.41	0.29	0.43	0.44	0.77
		beta	4.97	1.23	0.44	−1.44	0.82	−0.06
		95% CI	−0.02; 9.96	−1.72; 4.17	−0.40; 1.26	−5.02; 2.14	−1.27; 2.92	−0.48; 0.35
	Replication^a^	P value	7.05×10^−2^	3.55×10^−2^	0.94	0.25	0.45	0.94
		beta	2.26	1.69	0.01	1.11	0.47	0.01
		95% CI	−0.19; 4.71	0.11; 3.26	−0.34; 0.32	−0.79; 3.02	−0.77; 1.71	−0.21; 0.23
	KORA S3 and	P value	9.80×10^−3^	1.61×10^−2^	0.96	7.71×10^−2^	0.30	0.78
	replication^b^	beta	2.94	1.71	0.01	1.60	0.56	0.03
		95% CI	0.71; 5.17	0.32; 3.11	−0.34; 0.37	−0.17; 3.38	−0.50; 1.63	−0.18; 0.24
*Stratified by smoking* c *:*								
Smokers at the	KORA S3	P value	1.32×10^−2^	3.40×10^−2^	0.13	7.78×10^−2^	9.16×10^−4^	6.36×10^−2^
recruitment and/or		beta	5.58	2.99	0.46	2.95	3.45	0.41
previous regular		95% CI	1.18; 9.98	0.23; 5.74	−0.14; 1.06	−0.32; 6.21	1.42; 5.47	−0.02; 0.85
smoking periods	Replication^a^	P value	0.48	7.94×10^−2^	4.58×10^−2^	0.45	0.28	2.45×10^−2^
		beta	0.97	1.45	0.36	0.84	0.74	0.35
		95% CI	−1.69; 3.62	−0.17; 3.07	0.01; 0.71	−1.35; 3.04	−0.6; 2.08	0.04; 0.65
	KORA S3 and	P value	5.81×10^−2^	9.63×10^−3^	1.31×10^−2^	0.11	6.03×10^−3^	3.66×10^−3^
	replication^b^	beta	2.20	1.84	0.38	1.50	1.57	0.37
		95% CI	−0.08; 4.47	0.45; 3.24	0.08; 0.68	−0.32; 3.32	0.45; 2.68	0.12; 0.62
nonsmokers	KORA S3	P value	1.07×10^−3^	4.44×10^−2^	8.78×10^−4^	0.19	9.32×10^−2^	8.47×10^−2^
		beta	7.59	2.72	1.05	2.25	1.69	0.40
		95% CI	3.07; 12.11	0.07; 5.37	0.43; 1.68	−1.14; 5.64	−0.28; 3.65	−0.05; 0.85
	Replication^a^	P value	0.83	0.95	0.26	0.20	0.56	0.68
		beta	−0.34	0.07	0.19	1.59	0.42	−0.06
		95% CI	−3.53; 2.85	−1.81; 1.94	−0.14; 0.53	−0.83; 4.01	−1.00; 1.85	−0.34; 0.22
	KORA S3 and	P value	0.08	0.22	1.02×10^−2^	7.15×10^−2^	0.14	0.57
	replication^b^	beta	2.29	0.95	0.39	1.81	0.86	0.07
		95% CI	−0.31; 4.9	−0.58; 2.48	0.09; 0.68	−0.16; 3.78	−0.29; 2.01	−0.17; 0.31
*Stratified by alcohol*	*intake* c *:*							
high alcohol	KORA S3	P value	9.81×10^−4^	1.59×10^−2^	2.43×10^−3^	3.87×10^−3^	3.49×10^−3^	2.23×10^−2^
consumption		beta	8.91	4.18	1.17	5.92	3.82	0.60
(≥20 g/day)		95% CI	3.66; 14.17	0.80; 7.56	0.41; 1.92	1.93; 9.91	1.27; 6.36	0.09; 1.11
	Replication^a^	P value	0.31	8.94×10^−2^	7.05×10^−2^	0.24	0.22	0.47
		beta	1.62	1.64	0.35	1.51	0.96	0.13
		95% CI	−1.5; 4.74	−0.25; 3.54	−0.03; 0.72	−1.03; 4.05	−0.58; 2.51	−0.22; 0.48
	KORA S3 and	P value	1.00×10^−2^	7.66×10^−3^	2.98×10^−3^	1.09×10^−2^	1.01×10^−2^	0.06
	replication^b^	beta	3.53	2.25	0.51	2.79	1.74	0.28
		95% CI	0.84; 6.21	0.60; 3.90	0.17; 0.85	0.64; 4.93	0.41; 3.06	−0.01; 0.57
low alcohol	KORA S3	P value	1.11×10^−2^	7.84×10^−2^	5.28×10^−2^	0.41	1.34×10^−2^	0.10
consumption		beta	5.08	2.08	0.53	1.22	2.15	0.34
(<20 g/day)		95% CI	1.17; 8.98	−0.23; 4.39	−0.01; 1.06	−1.68; 4.12	0.45; 3.85	−0.07; 0.74
	Replication^a^	P value	0.85	0.66	0.14	0.47	0.54	0.32
		beta	−0.25	0.36	0.24	0.78	0.39	0.13
		95% CI	−2.94; 2.44	−1.24; 1.97	−0.08; 0.56	−1.31; 2.86	−0.85; 1.63	−0.13; 0.39
	KORA S3 and	P value	0.20	0.17	2.32×10^−2^	0.28	5.03×10^−2^	8.65×10^−2^
	replication^b^	beta	1.46	0.92	0.32	0.93	1.00	0.19
		95% CI	−0.75; 3.68	−0.4; 2.24	0.04; 0.59	−0.76; 2.62	0.00; 2.00	−0.03; 0.41

Associations were assessed using logistic (HYP) and linear (SBP, DBP) regressions under additive model with age and sex as covariates. Meta-analysis was performed using inverse variance method with fixed effect model. Beta – effect size; CI, confidence interval.

a meta-analysis of replication samples: KORA S4, HYPEST for SBP and DBP (n = 2,621); KORA S4, HYPEST, BRIGHT for HYP (n = 6,593).

b meta-analysis: KORA S3, KORA S4, HYPEST for SBP and DBP (n = 3,638); KORA S3, KORA S4, HYPEST, BRIGHT for HYP (n = 7,553).

c for the BRIGHT study participants the data of smoking habits and alcohol consumption was not available.

### Contribution lifestyle factors to genetic associations

**Figure 4:**
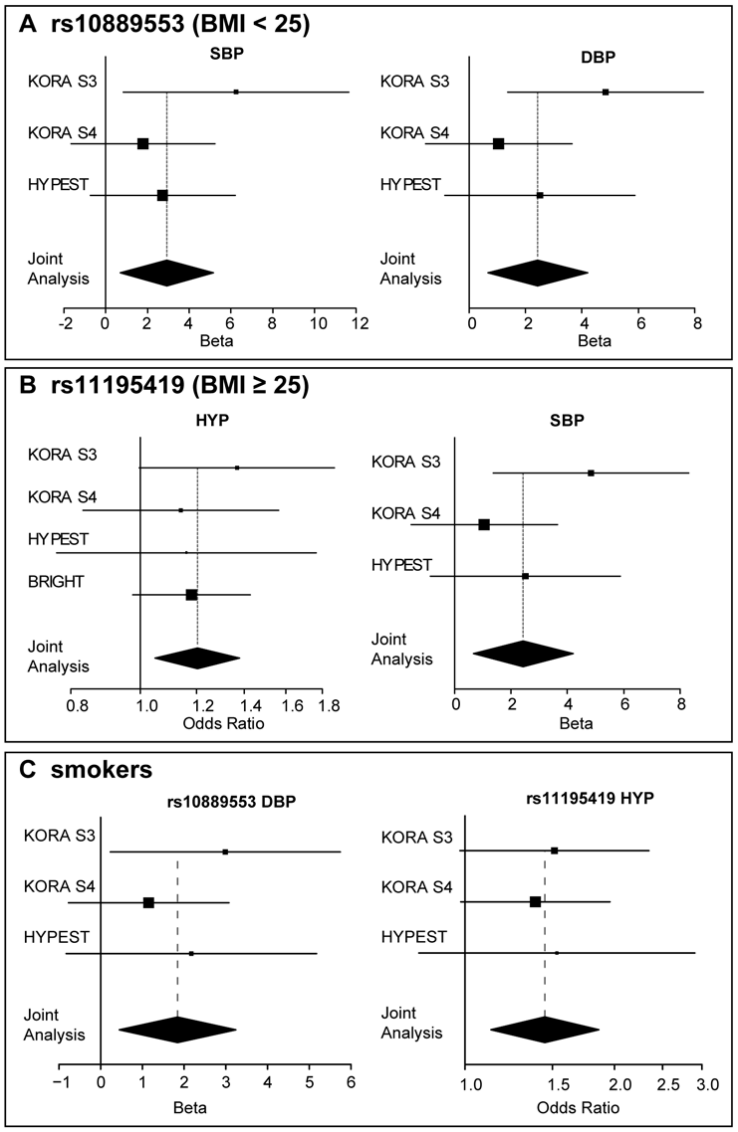
SNP effects in meta-analysis. Forest plots showing the effect sizes of the association of rs10889553 and rs11195419 with blood pressure in subjects stratified according to their Body Mass Index, BMI (<25 kg/m^2^ normal weight; ≥25 kg/m^2^ overweight) and smoking habits. Effects in individual populations KORA S3, KORA S4, HYPEST and BRIGHT are indicated by solid rectangles with sizes proportional to 95% confidence interval (horizontal lines). Solid diamonds represent 95% confidence intervals in meta-analysis using inverse variance method with fixed effects. Effects of association of (A) rs10889553 (*Leptin receptor, LEPR*) with systolic (SBP) and diastolic (DBP) blood pressure in normal weight population; (B) rs11195419 (*Alpha-2A-Adrenergic Receptor*, *ADRA2A*) in with hypertension (HYP) and SBP in overweight population; (C) rs10889553 with DBP and rs11195419 with HYP among smokers, including smokers at the recruitment and/or previous regular smokers during their lifetime.

Several metabolic and life-style parameters are known to affect blood pressure in humans. We explored the effects of body mass index (BMI), habits of smoking and alcohol consumption on the associations between blood pressure and seven genetic markers selected to the replication steps. We reanalyzed the discovery sample KORA S3 and the data from replication steps by subdividing individuals into normal weight (BMI<25 kg/m^2^) and overweight (BMI≥25 kg/m^2^) groups (**Table 3**). Similarly, the study samples were stratified based on their (i) smoking habits as non-smokers and smokers (including current and ex-smokers) as well as (ii) alcohol consumption as low consumers (<20 g/day) or high consumers (≥20 g/day). After subdivision of study samples based on BMI, the association of rs11195419 (*ADRA2A*) with hypertension was detected only in the overweight group (KORA S3: *P* = 5.21×10^−2^, OR = 1.37; Replication: *P* = 4.48×10^−2^, OR = 1.17; Meta-analysis: *P* = 8.0×10^−3^, OR = 1.20) and the individuals with normal weight were not affected (*P* = 0.78) (**Figure 4** **,** **Table 3**). The same SNP was associated with hypertension only among smokers (KORA S3: *P* = 6.36^−2^, OR = 1.51; Replication: *P* = 2.45×10^−2^, OR = 1.42; Meta-analysis: *P* = 3.66×10^−3^, OR = 1.45) and no effect was detected among non-smokers (*P* = 0.57).

In contrast, rs10889553 (*LEPR*) revealed association with SBP (KORA S3: *P* = 5.16×10^−2^, beta = 4.97; Replication: *P* = 7.05×10^−2^, beta = 2.26; Meta-analysis: *P* = 9.80×10^−3^, beta = 2.94;) and DBP (KORA S3: *P* = 4.1×10^−1^, beta = 1.23; Replication: *P* = 3.55×10^−2^, beta = 1.69; Meta-analysis: *P* = 1.61×10^−2^, effect = 1.71) only in the normal weight sub-group (**Figure 4** **,** **Table 3**). This genetic marker showed also accumulating evidence for the interaction with life-style factors. The association with DBP and HYP was detected among smokers (meta-analysis for DBP: *P* = 9.63×10^−3^, effect = 1.45; meta-analysis for HYP: *P* = 1.31×10^−2^, OR = 1.46) and high alcohol consumers (meta-analysis for DBP: *P* = 7.66×10^−3^, effect = 2.25; meta-analysis for HYP: *P* = 2.98×10^−3^, OR = 1.67). Non-smokers and low-alcohol consumers revealed no effect of the carrier status of rs10889553 genotype on their blood pressure (*P*>0.05). The remaining six SNPs selected for the replication did not show consistent and replicable interaction with studied life-style factors (**Table S5** **,** **Table S6** **,** **Table S7**).

### Haplotype analysis

**Figure 5:**
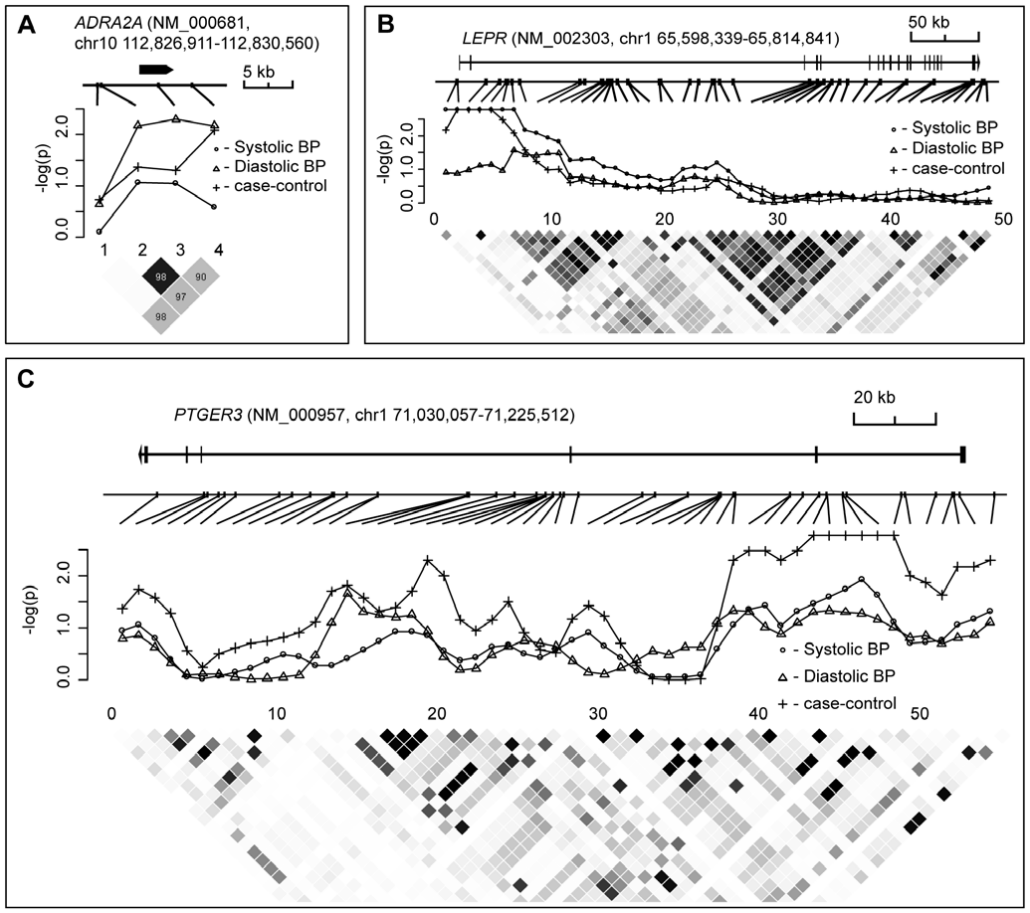
Results of haplotype association analysis with hypertension, SBP and DBP (detailed in Table 4). The upper part of each figure shows the exon-intron structure of the analyzed candidate gene along with the distribution of Affymetrix 500K SNPs in the locus. The middle and the lower part of the figures represent plotted *P*-values from the haplotype association tests under sliding window analysis (WHAP 2.09 software) and LD structure (R^2^) in KORA S3 study population, respectively. (A) *Alpha-2A-Adrenergic Receptor* (*ADRA2A*; sliding window = 1 marker); (B) *Leptin receptor* (*LEPR*, sliding window = 5 markers); (C) *Prostaglandin E Receptor* (*PTGER3*, sliding window = 3 markers).

We performed haplotype analysis for the seven genomic regions (*ADRA2A, LEP, LEPR, PTGER3, SLC2A1, SLC4A2, SLC8A1*), which revealed evidence for the association with blood pressure traits using single-marker tests (*P*<10^−3^) in the initial KORA S3 sample (**Table 1**). Haplotype association with HYP, SBP and DBP was tested using a regression-based method, which simultaneously performs phasing and also accounts for ambiguity in haplotype inference [17]. For each analysis based on the sliding window approach the tested haplotype length was defined by the number of neighboring SNPs forming a haplotype: one (for comparison with single SNP tests), three, five and eight SNPs. *ADRA2A*, *LEP*, *SLC2A1*, *SLC4A2* and *SLC8A1* loci displayed no haplotype effects as the strongest *P-*values from regression-based omnibus tests were obtained for window size = 1 (**Table 4** **,** **Figure 5A**). In two loci (*PTGER3* and *LEPR*) the haplotype analysis increased the strength of the associations. The best fitting window size for the association of *LEPR* haplotypes with HYP and SBP was five neighboring SNPs. For the *PTGER3* gene the strongest support was obtained for the association of three-marker haplotypes with HYP. Both, in *LEPR* and in *PTGER3* the region revealing strongest association with blood pressure is located in the 5′ part of the genes exhibiting moderate linkage disequilibrium (LD) and covering first exons and introns (**Figure 5B, C**).

**Table 4:** *P-*values of gene haplotype association tests using sliding window analysis and alternative haplotype lengths.

GENE (no of SNPs) Phenotype	WS*	ADRA2A (4)	LEP (16)	LEPR (49)	PTGER3 (55)	SLC2A1 (12)	SLC4A2 (8)	SLC8A1 (123)
Hypertension	1	**3.49×10^−2^**	0.103	1.83×10^−2^	3.16×10^−2^	0.582	**9.98×10^−3^**	**1.83×10^−2^**
	3	0.565	0.241	2.00×10^−2^	**3.33×10^−3^**	0.188	3.83×10^−2^	2.83×10^−2^
	5	na	0.183	**8.32×10^−3^**	4.99×10^−3^	0.436	0.135	5.32×10^−2^
	8	na	0.163	6.16×10^−2^	9.98×10^−3^	0.466	0.233	0.141
SBP	1	0.241	0.463	6.66×10^−3^	0.429	0.611	0.369	**1.50×10^−2^**
	3	0.296	0.587	1.33×10^−2^	0.195	0.195	0.351	4.33×10^−2^
	5	na	0.707	**1.66×10^−3^**	0.331	0.281	0.419	5.66×10^−2^
	8	na	0.606	3.33×10^−3^	0.366	0.278	0.381	8.65×10^−2^
DBP	1	**4.99×10^−3^**	0.749	0.121	0.215	**1.83×10^−2^**	0.642	7.99×10^−2^
	3	9.98×10^−3^	0.710	0.363	0.215	3.66×10^−2^	0.682	0.271
	5	na	0.501	0.141	0.253	6.49×10^−2^	0.634	0.423
	8	na	**8.65×10^−2^**	0.226	0.225	0.161	0.474	0.103

Analysis was conducted for the hypertension candidate genes revealing strongest single marker associations with hypertension, systolic (SBP) or diastolic (DBP) blood pressure. The lowest *P* values obtained for each gene are indicated in bold.

* Sliding window size - number of neighboring SNPs in the sliding window; na – not applicable.

**Figure 6:**
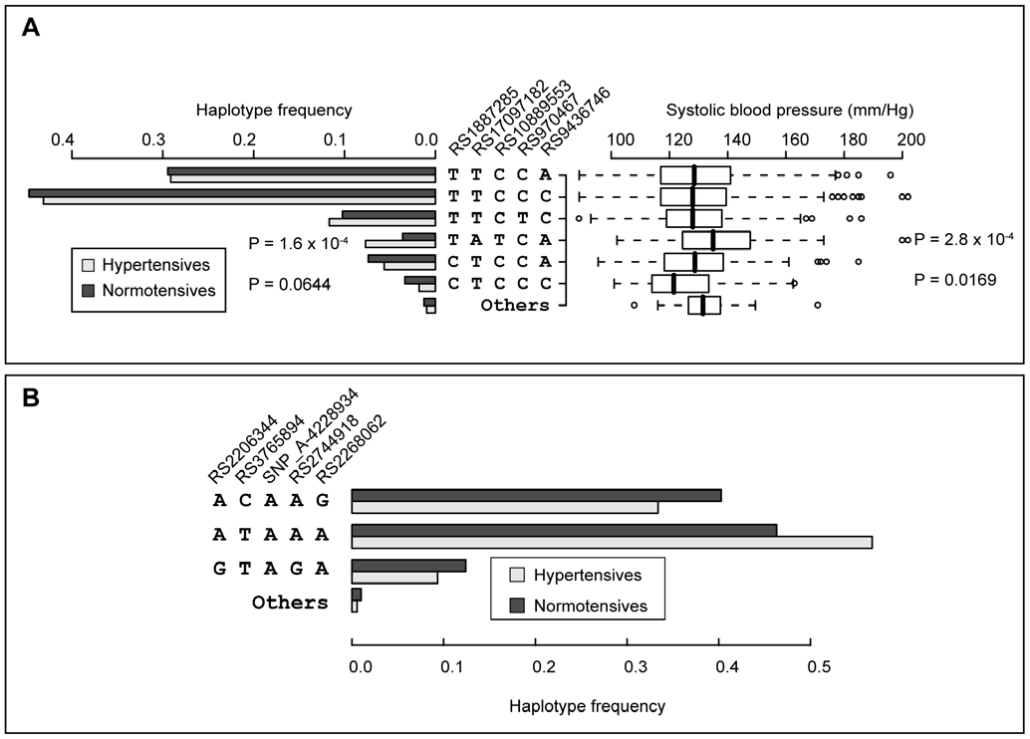
Effects of individual haplotypes of *Leptin receptor* (*LEPR*) and *Prostaglandin E Receptor* (*PTGER3*) genes on blood pressure traits. Rare haplotypes (frequency<1%) were pooled and are indicated as ‘Others’. Effect of gene haplotype on the distribution of SBP was tested by Mann-Whitney U-test (two-tailed); association of common haplotypes with hypertension was assessed by logistic regression. (A) *LEPR* gene, haplotypes combining rs1887285, rs17097182, rs10889553, rs970467, rs9436746. TATCA variant is associated with hypertension and higher systolic blood pressure, CTCCC variant is associated lower SBP and is more frequent in normotensives. (B) *PTGER3* gene, haplotypes combining rs2206344, rs3765894, SNP_A-4228934, rs2744918, rs2268062. ACAAG and GTAGA variants are more prevalent among normotensives, ATAAA haplotype is enriched among hypertensives.

In order to test the susceptibility of each gene variant to blood pressure determination, individual haplotypes of the 5′ part of *LEPR* and *PTGER3* were determined by *in silico* phasing (**Figure 6**). In the *LEPR* gene the haplotype TATCA (rs1887285, rs17097182, rs10889553, rs970467, rs9436746) was associated with hypertension (*P* = 1.7×10^−4^, OR = 2.19; hypertensives 8%; normotensives 4%) and higher SBP (*P* = 2.8×10^−4^, Mann-Whitney test) (**Figure 6A**). In contrast, the CTCCC variant was associated with lower SBP (*P* = 0.016) and was more frequent in normotensives (*P* = 0.064). Interestingly, the most common *PTGER3* haplotype ATAAA (rs2206344, rs3765894, SNP_A-4228934, rs2744918, rs2268062) was associated with the risk for hypertension (*P* = 1.6×10^−5^, OR = 1.50; hypertensives 57%; normotensives 46%), whereas two other prevalent variants exhibit a protective effect (**Figure 6B**).

## Discussion

In recent years genotyping technologies designed for genome-wide association (GWA) studies have started to offer simultaneous genotyping of large numbers of markers across many genes or genomic regions [23]. We utilized a subset of information from a genome wide genotyping dataset to address specifically the regions in the genome that have been previously identified to be involved in blood pressure (BP) regulation. The candidate gene targeted analysis allowed the combination of cost effectiveness with a hypothesis-based approach. In agreement with the fact that almost half of the BP candidate genes (46%) were insufficiently covered on the Affymetrix 500 k chip, we observed no associations in 126 (79%) of the studied genes. For many genes this result may indeed reflect the lack of contribution of these SNP markers to the susceptibility of hypertension and BP levels in the general population from Germany. However, there is no basis to generalize this across all populations as several susceptibility variants for hypertension have been shown to exhibit geographic region or population-specific associations. The lack of associations in a large proportion of genes can also be attributed to inadequate marker coverage on the Affymetrix 500 k chip. This may also have affected the low representation of the BP loci with prior functional evidence among the top results in previous GWA studies.

We explored the landscape of the genetic associations of the Affymetrix 500 k markers covering BP genes from several angles: single marker analysis followed by replication and meta-analysis, single-marker analysis of the study samples stratified by life-style factors (BMI, smoking and alcohol consumption), as well as haplotype analysis across top loci. In single marker analysis only 0.50% of the tested SNPs reached considerable association (*P*<10^−3^) with BP traits. The top SNPs were replicated in joint analysis of the discovery sample and replication resources. Interestingly, the two top SNPs of this study, rs11195419 (*ADRA2A*) and rs10889553 (*LEPR*) appeared to be consistently associated with BP traits in study samples stratified based on the body mass index (BMI) or life-style habits of the participants. rs11195419 (*ADRA2A*) revealed contribution to BP levels among overweight (BMI ≥25 kg/m^2^) subjects and smokers, whereas rs10889553 (*LEPR*) was associated with BP determination among normal weight population (BMI<25 kg/m^2^), smokers and high-alcohol consumers. This indicates that one should be careful in drawing conclusions about the contribution of the genetic variation in BP candidate genes based on GWA results of single studies. The variation of the study locus may not be well captured, the true effect of the particular polymorphism may be small and thus, may require a large sample size to be detected. Alternatively, the association with blood pressure traits may depend on other interacting factors such as BMI, smoking and alcohol consumption habits, age, health status etc. The challenge to identify blood pressure associations was further highlighted by power analysis. The discovery sample KORA S3 was sufficiently powered to identify associated markers with strong allelic effects (≥4 mmHg) and moderate to high minor allele frequencies (MAF>10%). Retrospective power calculations indicated that the detection of the two top associations in *LEPR* (rs10889553; MAF = 5.4%; effect on SBP in meta-analysis: 2.25 mmHg) and *ADRA2A* (rs11195419; MAF = 10.6%; effect on SBP in meta-analysis: 1.12 mmHg) at the Bonferroni significance level would have required sample sizes of 7,500 and 15,000–16,000 of the discovery sample, respectively.

Even though most of the markers in this study did not gain statistical support for the contribution to the BP traits, some of the most interesting associations are warrant further consideration. In the initial evidence based association study with KORA S3 two of the strongest associations were identified with markers in *leptin* (*LEP*; 7q32.1) and *leptin receptor* (*LEPR*; 1p31.3) genes, components of a single regulatory pathway. However, the study lacked the power to attempt replication of rs10954174 in *leptin* as its minor allele frequency is only 0.1%. In literature, another rare *LEP* SNP located ∼1.7 kb downstream from rs10954174 in the *3′ UTR* has been associated with pulse pressure [24]. The support for the association of *LEPR* with blood pressure was obtained by several alternative approaches: in the discovery sample with all three BP traits (*P*≤3.16×10^−3^), in meta-analysis across all cohort samples, in haplotype analysis and in life-style stratified subgroup analysis. rs10889553 is located in the second intron of the *LEPR* gene, upstream of the coding region starting with exon 3. Previously, exonic polymorphisms in *LEPR* have been associated with hypertension in obesity [25], [26]. It has been shown that the first introns may contain regulatory elements and may be involved in transcriptional regulation [27]. For example, a GWAS for fat mass and obesity identified the strongest association in the first intron of *FTO* gene [28]. Although initially the *LEPR* gene and its variants have been implicated in the determination of body mass and related phenotypes [29], there is growing evidence about the wider role of *LEPR* in human metabolism. Recent studies have associated *LEPR* gene variants with inflammatory traits like plasma fibrinogen and C-reactive protein levels [30], bone density [31], [32] and insulin resistance in nondiabetic obese patients [33].

The second top SNP (rs11195419) highlighted in this study is located within *3′ UTR* of *Alpha-2A-Adrenergic Receptor (ADRA2A*; 10q25.2*)* and was associated with blood pressure and hypertension in overweight individuals (BMI≥25 kg/m^2^) and among smokers. *ADRA2A* mediates stress responses and cardiac regulation via the sympathetic nervous system and is also involved in the inhibition of fatty acid mobilization from adipose tissue [34]. Consistently with our results, a *DraI* RFLP in the *3′ UTR* of *ADRA2A* and an upstream polymorphism *C-1291G* have been associated with adiposity and abdominal obesity [34], [35], as well as with increased hypertension prevalence [36]–[38].

Our results suggest that environmental and life-style factors may influence the penetrance of genetic risk factors for hypertension. For example, inherited risk alleles for high blood pressure in the *LEPR* gene affect the onset of hypertension predominantly in individuals with unhealthy life-style. The results of this study may also have implications for anti-hypertensive therapy. As the gene *ADRA2A* codes for one of the targets for alpha adrenoreceptor antagonists (also called alpha-blockers), the response to the treatment may depend on carrier status of the identified gene variants in conjunction with smoking habits.

Haplotype analysis may identify susceptibility loci that are not captured by single marker tests and/or reveal the combinatory effect of loci [23]. We identified haplotypes of *LEPR*, but also *PTGER3* gene, which were associated with increased risk for hypertension in KORA S3 study population. *PTGER3* is coding for the receptor for prostaglandin E2, which may be involved in a variety of functions including modulation of neurotransmitter release in central and peripheral neurons and inhibition of sodium and water reabsorption in kidney tubulus [39]. To our knowledge, no studies have been conducted so far targeting the contribution of *PTGER3* gene variants to the determination of blood pressure.

In summary, using genome-wide genotyping array for targeting candidate genes for a complex disease needs prior knowledge of the SNP coverage on the genotyping array of choice. In some genomic regions with sufficient LD, imputation could be one way to overcome the low SNP density [40]. However, the major limiting factor in blood pressure association studies appears to be the small phenotypic effect of causal loci and complex interaction with life-style, general health-status and age-associated metabolic parameters. Even though in this study two genes, *LEPR* and *ADRA2A* appeared to display supportive evidence for association with blood pressure across different populations, further studies are required to draw clear conclusions about their contribution to the determination of blood pressure.

## Supporting Information

Table S1 List of markers with association test p values<10^−2^. ^a^ Coordinates include genic region ±10 kb. Coordinates refer to human genome build 35; ^b^% of SNPs (MAF≥0.05) in Hapmap phase II (CEU population) localized in genic regions that were tagged on the Affymetrix 500 k chip with r^2^≥0.8. The analysis included SNPs on the Affimetrix array within ±200 kb from a gene and passing quality control criteria in the KORA 500K genotyping. ^c^SBP - Systolic Blood pressure; DBP - Diastolic blood pressure; Hypertension - Hypertension case-control; ^d^Statistical tests were performed with plink software.
(0.04 MB XLS)

Table S2 List of candidate regions with no significant associations. ^a^Coordinates include genic region ±10 kb. Coordinates refer to human genome build 35; ^b^% of SNPs (MAF≥0.05) in Hapmap phase II (CEU population) localized in genic regions that were tagged on the Affymetrix 500 k chip with r^2^≥0.8. The analysis included SNPs on the Affimetrix array within ±200 kb from a gene and passing quality control criteria in the KORA 500K genotyping.
(0.09 MB XLS)

Table S3 Demographics of the studied population samples.
(0.01 MB XLS)

Table S4 Association results with SNPs entering replication in KORA S4. Results of regression analyses with age and sex as covariates.
(0.01 MB XLS)

Table S5 Association results with SNPs entering replication performed in study samples stratified according to BMI of the participants. Results of regression analyses with age and sex as covariates. Meta analysis over indicated populations was performed using inverse variance method with a fixed effect model
(0.02 MB XLS)

Table S6 Association results with SNPs entering replication performed in study samples stratified according to smoking habits of the participants. Results of regression analyses with age and sex as covariates. Meta analysis over indicated populations was performed using inverse variance method with a fixed effect model. Non-smokers - never smoked. Smokers - smokers at the recruitment and/or previously during lifetime.
(0.02 MB XLS)

Table S7 Association results with SNPs entering replication performed in study samples stratified according to alcohol consumption habits of the participants. Results of regression analyses with age and sex as covariates. Meta analysis over indicated populations was performed using inverse variance method with a fixed effect model.
(0.02 MB XLS)
